# Different Trends in the Incidence and Mortality Rates of Prostate Cancer Between China and the USA: A Joinpoint and Age-Period-Cohort Analysis

**DOI:** 10.3389/fmed.2022.824464

**Published:** 2022-02-03

**Authors:** Hairong He, Liang Liang, Didi Han, Fengshuo Xu, Jun Lyu

**Affiliations:** ^1^Clinical Research Center, The First Affiliated Hospital of Xi'an Jiaotong University, Xi'an, China; ^2^School of Public Health, Xi'an Jiaotong University Health Science Center, Xi'an, China; ^3^Department of Urology, The First Affiliated Hospital of Xi'an Jiaotong University, Xi'an, China; ^4^Department of Clinical Research, The First Affiliated Hospital of Jinan University, Guangzhou, China

**Keywords:** prostate cancer, incidence, death, China, USA

## Abstract

**Purpose:**

This study used data from the Global Burden of Disease Study 2019 (GBD 2019) to determine the differences in the incidence and mortality of prostate cancer (PCa) between China and the USA from 1990 to 2019.

**Method:**

The age-standardized incidence rates (ASIRs) and age-standardized death rates (ASDRs) in China and the USA from 1990 to 2019 were extracted from GBD 2019. Annual percentage changes and relative risks of ASIR and ASDR were calculated using joinpoint regression analysis and age-period-cohort models, respectively.

**Results:**

The ASIR of PCa in China continually increased from 1990 to 2019, while in the USA it increased from 1990 to 1994 and then continually decreased until 2015, and then slightly increased again until 2019. The ASDR in China did not change, and the trend of ASDR in the USA was similar to the trend of the ASIR in the USA. The incidence of PCa increased with age in China, but decreased after the age of 75 years in the USA. A period effect was present, with the risk of developing PCa increasing continuously over longer time periods. Those born later had a lower risk of PCa or death, indicating a cohort effect.

**Conclusion:**

PCa is becoming more problematic for Chinese males. Disease trends in the USA indicate that large-scale screening may be beneficial and should be immediately implemented among high-risk groups in China.

## Introduction

In 2017, prostate cancer (PCa) was the most common cancer among males worldwide, with 1.7 million new cases, and its incidence has continually increased recently (1). The incidence, characteristics at onset (e.g., severity of disease and age at onset), and incidence trend of PCa vary markedly between countries (2). The reasons for this include differences in the implementation of PCa screening and its policies, the specific risk genotypes of different races, and diet (3). In addition, differences in morbidity and clinical features have also created huge differences in mortality between countries.

Understanding the differences in the changing trends of PCa burden between countries could identify favorable policy recommendations. In this context, the USA may be the most useful reference since it has generally provided routine PCa screening to those older than 50 years since the 1990s (4). After decades of practice, the screening policy has been adjusted multiple times, which has had an impact on the incidence and mortality trends of PCa (5, 6). In contrast, China does not have a national policy for PCa screening. In addition, China is encountering formidable healthcare challenges brought about by the problem of aging (7), coupled with increasingly westernized diet, resulting in the estimated incidence of PCa in China being increasing (8), which suggested the need for targeted screening programs.

PCa is a disease that is greatly affected by age and the environment (9). Understanding these effects will improve the understanding of the epidemiological risk factors for PCa, which is beneficial for disease control and management. The age-period-cohort model can determine the impacts on PCa incidence and mortality of different ages, periods, and birth cohorts, with extrapolation used to estimate the impact of different age brackets, a complex set of historical events and environmental factors, and generational characteristics including risk factors and exposure to environmental factors in early life, respectively (10). Meanwhile, this model also helps in identifying high-risk groups that need intervention and management.

The present study aimed to compare the trends in the incidence and mortality of PCa in China and the USA from 1990 to 2019, and establish age-period-cohort models for the two countries based on data from the Global Burden of Disease Study 2019 (GBD 2019). The obtained results may help to increase the understanding of the epidemiological characteristics and environmental effects of PCa in China, with comparisons with the USA used to provide evidence for the prevention and management of PCa in China.

## Materials and Methods

### Data Source

The data used in this study were from GBD 2019. We extracted the age-standardize incidence rates (ASIRs) and age-standardized death rates (ASDRs) in China and the USA from 1990 to 2019. Additionally, to analyze the effects of age on incidence and mortality rates, we also extracted the data of the 12 different age groups used in GBD 2019, comprising 11 5-year periods from 40 to 94 years, and ≥95 years.

The age-standardize rates were calculated by summing up the products of the age-specific rates (*a*_*i*_, where *i* is the *i*th age class), and the number of persons (or the weight) (*w*_*i*_) in the same age subgroup i of the selected reference standard population, then dividing the sum of the standard population weights: age-standardize rates $=\frac{\overset{A}{\underset{i=1}{\sum }}a_{i}w_{i}}{\overset{A}{\underset{i=1}{\sum }}w_{i}}\times 100,000$. Namely, the ASIR corresponds to the number of cases per 100,000 persons, and the ASDR corresponds to the death number per 100,000 persons after age standardization.

### Joinpoint Regression Analysis

The apparent long-term trends are important issues when analyzing disease incidence and mortality data. This study employed a joinpoint regression model (version 4.7.0, Joinpoint, IMS, Calverton, MD, USA) to determine the incidence and mortality rate trends of PCa. The basic principles of this model are to divide the long-term trend of epidemiology into multiple segments based on inflection points, and to create a straight line for each segment to describe the epidemiological trend of a certain disease over a certain time period (11). The annual percentage change (APC) and its corresponding 95% confidence interval (CI) are used to quantify the magnitude of each epidemiological trend.

The APC of each segment was calculated using a log-linear model according to APC = (*e*^β^1) × 100%, where β is the coefficient of the linear model (12). The statistical significance of APC was judged by testing whether β was 0, with a testing threshold of α = 0.05. When the APC value and its 95% CI exceeded 0, an increase in incidence or mortality of PCa was present during this period; when these values were <0, a decrease in incidence or mortality of PCa was present during this period; and when the *P*-value was > 0.05, no significant changes in PCa incidence or mortality occurred during this period. The average APC (AAPC) of the whole study periods was calculated by weighting the regression coefficient of each segment by the span width of the segmented interval.

### Age-Period-Cohort Analysis

The age-period-cohort model is a statistical analysis method commonly used in demographics, sociology, and epidemiology. The model can estimate the age, period, and cohort effects on trends in the incidence and mortality rates. The age-period-cohort model is based on the Poisson distribution (13):


$$\begin{array}{l} ln\left[\text{E}\left(M_{ij}\right)\right]=ln\left(D_{ij}/P_{ij}\right)=\mu +\alpha_{i}+\beta_{i}+\;\gamma_{k} \end{array}$$

where *E* represents the expected number of incident cases or deaths in the *i*-th age group and *j*-th period, and is assumed to conform to the Poisson distribution; *M*_*ij*_, *D*_*ij*_, and *P*_*ij*_ represent the incidence or mortality rate, the total number of incident or mortality cases, and population size in the (*i, j*) group, respectively; μ represents the intercept or adjusted average incidence or mortality rate; and α_*i*_, β_*j*_, and γ_*k*_ represent the coefficient of the age effect or the coefficient in the *i*-th age group, the period effect or the coefficient in the *j*-th period group, and the cohort effect or the coefficient in the *k*-th cohort group, respectively (where *k* = *i* + *j* – 1).

The data were organized based on the existing format of the GBD database. We used the same 17 age groups as GBD 2019, namely 20–24, 25–29, …, 90–94 years old. In order to avoid overlapping information between adjacent cohorts, the group interval of each period was every five years from 1990–2019, namely 1990–1994, 1995–1999, …, 2014–2019. Therefore, 20 cohorts were generated based on the 17 age groups and 6 period groups: 1904–1908, 1909–1913, …, 1999–2003.

Multicollinearity between age, period, and birth cohorts is inevitable. Any one of these three independent variables can be combined with the other two to become linear, which makes it difficult to estimate the unique set for every age, period, and cohort effect. To overcome this, the age-period-cohort framework with the intrinsic estimator method was used to account for the varying effects over the interrelated time scales of chronological age, diagnosis calendar period, and year of birth. The age-period-cohort framework with the intrinsic estimator method estimated coefficients of the age, period, and cohort effects. We then transformed coefficients into exponential values to determine the relative risks (RRs) for incidence and mortality rates of each age, period, or birth cohort relative to the average combined level of all ages, periods, or birth cohorts.

## Results

### Incidence and Mortality Rate Trends of PCa in China and the USA

**Figure 1:**
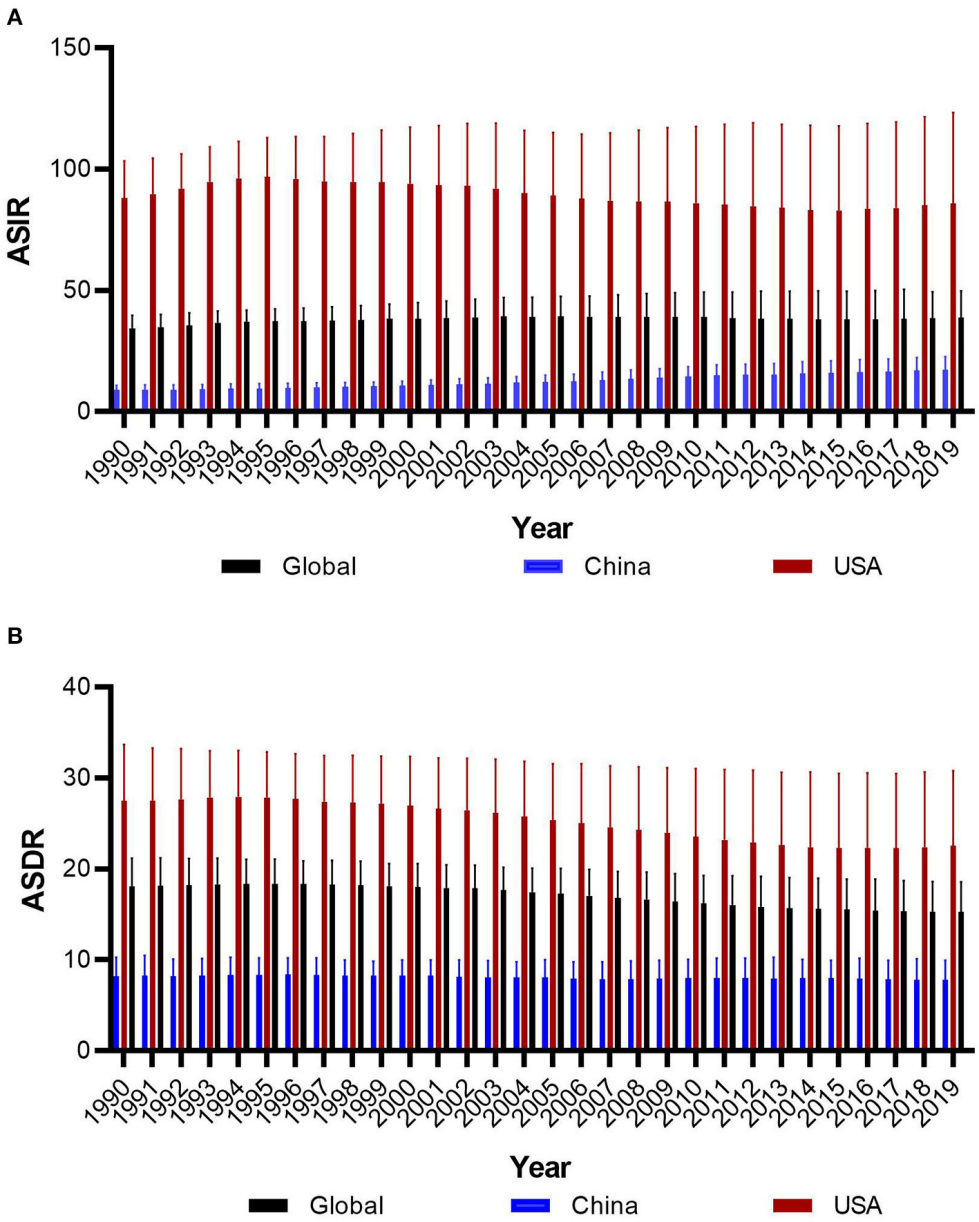
The trends of age-standard incidence rate **(A)** and death rate **(B)** of prostate cancer in global, China and America from 1990 to 2019.

**Figure 2:**
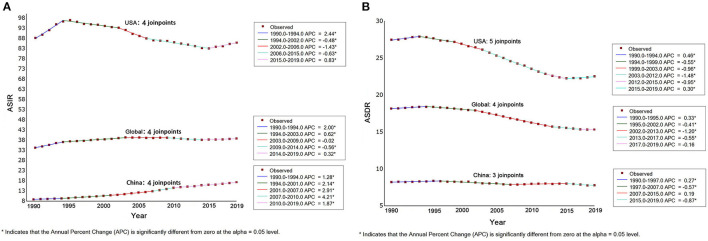
The the results of joinpoint regression analysis for age-standard incidence rate **(A)** and death rate **(B)** protate cancer in global, China and America from 1990 to 2019.

Figure 1 presents the ASIRs and ASDRs in China and the USA from 1990 to 2019, and Figure 2 presents the results of joinpoint regression analysis. The ASIRs in China and the USA in any given year were much lower and much higher than the global incidence rate, respectively. There was a continuously increasing trend for China, with ASIR being 8.88/100,000 persons in 1990 and 17.34/100,000 persons in 2019 (AAPC = 2.30, 95% CI = 2.10–2.50). The four joinpoints for China indicated that the increasing trend was most rapid from 2007 to 2010 (APC = 4.21, 95% CI = 2.48–5.97). Meanwhile, the trend for the USA was not stable after undergoing four joinpoints. The ASIR increased rapidly between 1990 and 1994, from 88.06/100,000 persons to 96.18/100,000 persons (APC = 2.44, 95% CI = 2.10–2.78). From 1994 to 2015, the ASIR decreased to 82.90/100,000 persons at varying rates (APC = −0.48, −1.43, and −0.63 for 1994–2002, 2002–2006, and 2006–2015, respectively). ASIR exhibited an increasing trend from 2015 to 2019, when it was 85.80/100,000 persons (APC = 0.83, 95% CI = 0.49–1.16).

The ASDRs in China and the USA in any given year were much lower and much higher than the global rate, respectively. The trend indicated differing patterns between China and the USA. There was a slightly decreasing general trend in China, from 8.22/100,000 persons in 1990 to 7.79/100,000 persons in 2019 (AAPC = −0.2, 95% CI = −0.3 to −0.1), and experienced three joinpoints of a slight increase from 1990 to 1997 (APC = 0.27, 95% CI = 0.07–0.48), a slight decrease from 1997 to 2007 (APC = −0.57, 95% CI = −0.71 to −0.43), a slight decrease from 2015 to 2019 (APC = −0.87, 95% CI = −1.35 to −0.39), and no changes from 2007 to 2015. Meanwhile, the trend for the USA was also not stable with five joinpoints, although the general trend of ASDR showed a decrease from 27.49/100,000 persons in 1990 to 22.39/100,000 persons in 2019 (AAPC = −0.7, 95% CI = −0.8 to −0.6). The trend showed increases from 1990 to 1994 and 2015 to 2019, and a decrease from 1994 to 2015.

### Incidence and Mortality Rates at Different Ages in China and the USA

**Figure 3:**
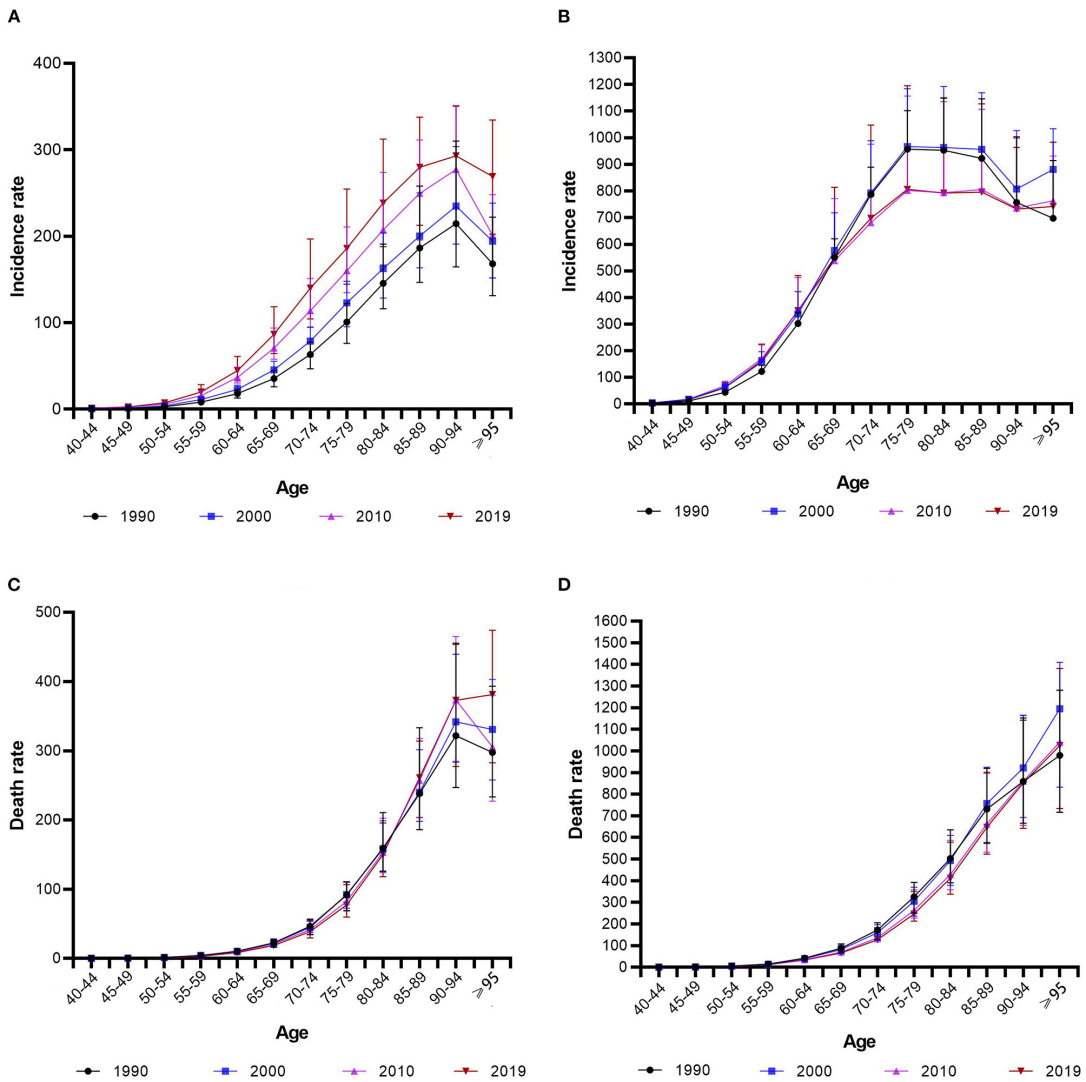
The incidence and mortality rates of protate cancer of different age groups for China [**(A)** incidence rate; **(C)** mortality rate] and the USA [**(B)** incidence rate; **(D)** mortality rate] in 1990, 2000, 2010, and 2019.

Figure 3 shows the incidence and mortality rates of different age groups for China and the USA in 1990, 2000, 2010, and 2019. In these four observation years, the PCa incidence in China increased with age between the ages of 40 and 94 years and then decreased at the age of ≥95 years. Meanwhile, the incidence in each age group also increased each year (Figure 3A). While, over the four observation years, the PCa incidence in the USA increased with age between the ages of 40 and 79 years and then did not change or slightly decreased up to the age of 94 years. Meanwhile, the PCa incidence over the four observation years in the USA were very similar for those aged 40 to 69 years, while the rates for those aged 70 to 89 years were lower in 2010 and 2019 than in 1990 and 2000 (Figure 3B).

Over the four observation years, the PCa mortality rate in China increased with age between the ages of 40 and 94 years. Meanwhile, the incidence was similar each year for those aged 40 to 84 years and was slightly higher in 2010 and 2019 than in 1990 and 2000 for those aged 85–89 and 90–94 years (Figure 3C). Meanwhile, over the four observation years, the PCa incidence in the USA increased with age. For those aged 70–89 years, the mortality rates were lower in 2010 and 2019 than in 1990 and 2000 (Figure 3D).

### Age-Period-Cohort Analysis

**Figure 4:**
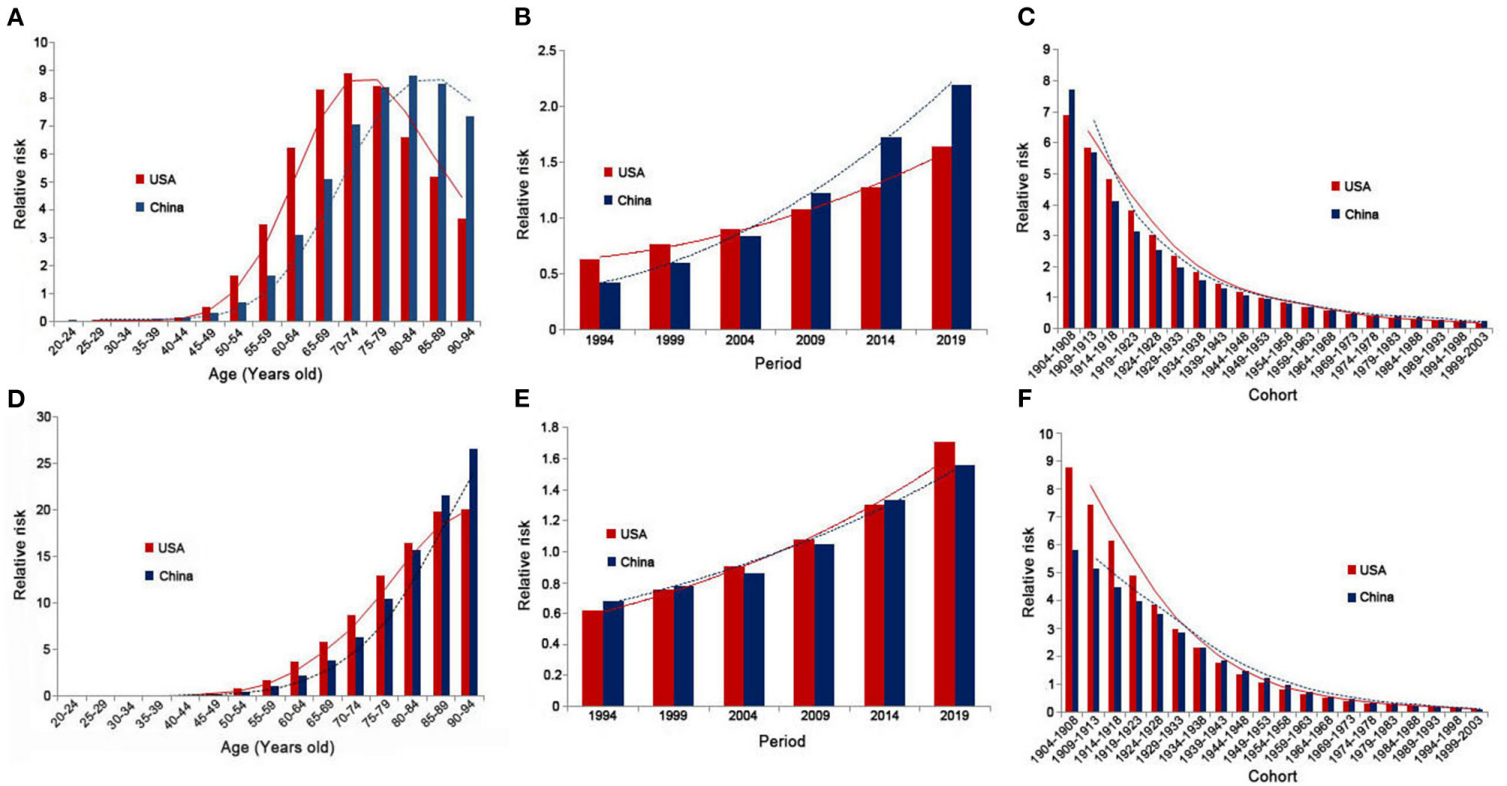
Relative risks of prostate cancer incidence and mortality rates in China and the USA from 1990 to 2019 due to age [**(A)** incidence rate; **(D)** mortality rate], period [**(B)** incidence rate; **(E)** mortality rate], and cohort [**(C)** incidence rate; **(F)** mortality rate] effects.

Table 1 lists the results of age-period-cohort analysis for incidence and mortality rates in China and the USA. Figure 4 shows relative risks of prostate cancer incidence and mortality rates in China and the USA from 1990 to 2019 due to age, period, and cohort effects. After controlling for the period and cohort effects, the age effect significantly impacted the PCa incidence rates for both China and the USA. The RR in those older than 55 years was higher than the average level of the total Chinese population, and was also higher for those older than 50 years in the USA compared with the total population. For China, the RR continuously increased from 0.065 (95% CI = 0.01–0.436) in those aged 20–24 years to 8.844 (95% CI = 7.137–10.96) in those aged 80–84 years, then decreased to 7.355 (95% CI = 5.107–10.594) in those aged 90–94 years. For the USA, the RR increased from 0.039 (95% CI = 0.012–0.119) in those aged 20–24 years to 8.915 (95% CI = 8.018–9.912) in those aged 70–74 years, then decreased to 3.685 (95% CI = 2.944–4.612) in those aged 90–94 years. The RR for the mortality rate of those older than 60 years was higher than the average level for the total Chinese and USA populations. The RR in China continuously increased from 2.172 (95% CI = 1.136–4.154) in those aged 60–64 years to 26.546 (95% CI = 11.078–63.61) in those aged 90–94 years. The RR in the USA continuously increased from 3.673 (95% CI = 2.203–6.122) in those aged 60–64 years to 20.07 (95% CI = 9.673–41.642) in those aged 90–94 years.

**Table 1:** The age, period and cohort effects on incidence and death rate in China and USA.

**Factors**	**Incidence rate**				**Death rate**			
	**China**		**USA**		**China**		**USA**	
	**RR (95% CI)**	***P***	**RR (95% CI)**	***P***	**RR (95% CI)**	***P***	**RR (95% CI)**	***P***
Age 20–24	0.065 (0.01–0.436)	0.005	0.039 (0.012–0.119)	<0.001	0.054 (0.001–3.005)	0.154	0.028 (0.001–1.131)	0.058
Age 25–29	0.072 (0.016–0.334)	0.001	0.045 (0.018–0.11)	<0.001	0.058 (0.002–1.541)	0.089	0.032 (0.002–0.607)	0.022
Age 30–34	0.083 (0.022–0.322)	<0.001	0.048 (0.021–0.109)	<0.001	0.07 (0.004–1.14)	0.062	0.037 (0.003–0.477)	0.012
Age 35–39	0.096 (0.029–0.324)	<0.001	0.058 (0.029–0.119)	<0.001	0.078 (0.007–0.909)	0.042	0.046 (0.005–0.399)	0.005
Age 40–44	0.142 (0.052–0.387)	<0.001	0.134 (0.082–0.22)	<0.001	0.113 (0.016–0.802)	0.029	0.099 (0.022–0.454)	0.003
Age 45–49	0.312 (0.147–0.662)	0.002	0.534 (0.381–0.747)	<0.001	0.217 (0.05–0.938)	0.041	0.304 (0.104–0.891)	0.03
Age 50–54	0.704 (0.391–1.267)	0.241	1.658 (1.274–2.157)	<0.001	0.454 (0.148–1.386)	0.165	0.806 (0.349–1.863)	0.614
Age 55–59	1.637 (1.034–2.592)	0.036	3.491 (2.83–4.306)	<0.001	1.052 (0.45–2.46)	0.906	1.699 (0.877–3.295)	0.116
Age 60–64	3.098 (2.166–4.431)	<0.001	6.233 (5.293–7.34)	<0.001	2.172 (1.136–4.154)	0.019	3.673 (2.203–6.122)	<0.001
Age 65–69	5.113 (3.9–6.704)	<0.001	8.322 (7.334–9.444)	<0.001	3.854 (2.343–6.34)	<0.001	5.835 (3.928–8.668)	<0.001
Age 70–74	7.085 (5.762–8.712)	<0.001	8.915 (8.018–9.912)	<0.001	6.346 (4.182–9.628)	<0.001	8.75 (6.248–12.254)	<0.001
Age 75–79	8.395 (6.984–10.091)	<0.001	8.445 (7.559–9.435)	<0.001	10.43 (6.742–16.136)	<0.001	12.981 (9.054–18.611)	<0.001
Age 80–84	8.844 (7.137–10.96)	<0.001	6.629 (5.772–7.613)	<0.001	15.701 (9.121–27.028)	<0.001	16.444 (10.452–25.872)	<0.001
Age 85–89	8.519 (6.432–11.282)	<0.001	5.212 (4.361–6.23)	<0.001	21.638 (10.774–43.457)	<0.001	19.823 (11.068–35.505)	<0.001
Age 90–94	7.355 (5.107–10.594)	<0.001	3.685 (2.944–4.612)	<0.001	26.546 (11.078–63.61)	<0.001	20.07 (9.673–41.642)	<0.001
Period 1994	0.427 (0.328–0.555)	<0.001	0.632 (0.552–0.724)	<0.001	0.68 (0.404–1.145)	0.147	0.619 (0.403–0.951)	0.029
Period 1999	0.604 (0.513–0.711)	<0.001	0.771 (0.709–0.838)	<0.001	0.781 (0.569–1.07)	0.124	0.751 (0.58–0.973)	0.03
Period 2004	0.836 (0.773–0.905)	<0.001	0.908 (0.876–0.942)	<0.001	0.863 (0.764–0.974)	0.017	0.9 (0.82–0.987)	0.026
Period 2009	1.226 (1.135–1.325)	<0.001	1.075 (1.036–1.116)	<0.001	1.05 (0.93–1.187)	0.429	1.075 (0.979–1.181)	0.129
Period 2014	1.726 (1.469–2.028)	<0.001	1.282 (1.178–1.394)	<0.001	1.336 (0.974–1.832)	0.072	1.301 (1.004–1.686)	0.047
Period 2019	2.192 (1.699–2.829)	<0.001	1.64 (1.433–1.877)	<0.001	1.556 (0.927–2.612)	0.094	1.707 (1.113–2.618)	0.014
Cohort 1904–1908	7.722 (4.393–13.575)	<0.001	6.898 (5.076–9.375)	<0.001	5.817 (1.859–18.207)	0.002	8.795 (3.355–23.056)	<0.001
Cohort 1909–1913	5.693 (3.585–9.041)	<0.001	5.835 (4.518–7.535)	<0.001	5.167 (1.979–13.493)	0.001	7.449 (3.297–16.832)	<0.001
Cohort 1914–1918	4.128 (2.849–5.983)	<0.001	4.836 (3.917–5.972)	<0.001	4.493 (2.029–9.948)	<0.001	6.168 (3.128–12.16)	<0.001
Cohort 1919–1923	3.148 (2.358–4.204)	<0.001	3.824 (3.218–4.543)	<0.001	3.99 (2.073–7.681)	<0.001	4.902 (2.794–8.602)	<0.001
Cohort 1924–1928	2.529 (2.019–3.166)	<0.001	3.024 (2.616–3.494)	<0.001	3.522 (2.016–6.155)	<0.001	3.86 (2.389–6.235)	<0.001
Cohort 1929–1933	1.967 (1.619–2.391)	<0.001	2.342 (2.049–2.677)	<0.001	2.859 (1.687–4.847)	<0.001	3.003 (1.916–4.708)	<0.001
Cohort 1934–1938	1.58 (1.269–1.967)	<0.001	1.818 (1.574–2.101)	<0.001	2.337 (1.311–4.167)	0.004	2.32 (1.431–3.762)	0.001
Cohort 1939–1943	1.302 (0.983–1.724)	0.065	1.441 (1.214–1.712)	<0.001	1.876 (0.94–3.745)	0.074	1.782 (1.009–3.149)	0.047
Cohort 1944–1948	1.082 (0.754–1.552)	0.67	1.183 (0.959–1.46)	0.116	1.49 (0.641–3.462)	0.354	1.361 (0.684–2.709)	0.38
Cohort 1949–1953	0.961 (0.612–1.509)	0.863	0.994 (0.771–1.281)	0.964	1.224 (0.442–3.39)	0.698	1.05 (0.459–2.402)	0.908
Cohort 1954–1958	0.814 (0.47–1.409)	0.462	0.846 (0.626–1.143)	0.276	0.964 (0.286–3.244)	0.952	0.828 (0.311–2.204)	0.705
Cohort 1959–1963	0.689 (0.358–1.324)	0.264	0.716 (0.503–1.017)	0.062	0.751 (0.179–3.148)	0.695	0.656 (0.209–2.059)	0.47
Cohort 1964–1968	0.587 (0.271–1.269)	0.176	0.587 (0.391–0.883)	0.01	0.586 (0.107–3.2)	0.538	0.517 (0.137–1.954)	0.331
Cohort 1969–1973	0.505 (0.202–1.262)	0.144	0.463 (0.288–0.743)	0.001	0.475 (0.063–3.612)	0.472	0.407 (0.086–1.916)	0.255
Cohort 1974–1978	0.426 (0.142–1.274)	0.127	0.379 (0.213–0.676)	0.001	0.377 (0.033–4.284)	0.432	0.326 (0.05–2.121)	0.241
Cohort 1979–1983	0.386 (0.102–1.454)	0.159	0.335 (0.152–0.738)	0.007	0.311 (0.015–6.496)	0.452	0.274 (0.023–3.307)	0.308
Cohort 1984–1988	0.347 (0.074–1.618)	0.178	0.296 (0.107–0.821)	0.019	0.251 (0.006–10.721)	0.47	0.223 (0.008–5.982)	0.371
Cohort 1989–1993	0.299 (0.049–1.842)	0.193	0.252 (0.073–0.873)	0.03	0.197 (0.002–21.279)	0.497	0.181 (0.003–11.167)	0.416
Cohort 1994–1998	0.253 (0.025–2.6)	0.248	0.217 (0.045–1.051)	0.058	0.153 (0–83.407)	0.559	0.149 (0.001–32.644)	0.489
Cohort 1999–2003	0.23 (0.005–10.315)	0.449	0.182 (0.012–2.65)	0.212	0.129 (0–5441.571)	0.706	0.12 (0–1189.473)	0.652
Deviance	4.63		19.378		4.566		0.951	
AIC	5.08		6.828		4.372		5.143	
BIC	229.36		−214.612		−229.424		−233.039	

*AIC, Akaike Information Criterion; BIC, Bayesian Information Criterion*.

After controlling for age and cohort effects, the period effect significantly impacted the PCa incidence rate in both China and the USA. The RRs in 2009, 2014, and 2019 were higher than the average levels in China and the USA, and were lower in 1994, 1999, and 2004. The RR in China continuously increased from 0.427 (95% CI = 0.328–0.555) in 1994 to 2.192 (95% CI = 1.699–2.829) in 2019, and that in the USA increased from 0.632 (95% CI = 0.552–0.724) in 1994 to 1.64 (95% CI = 1.433–1.877) in 2019. The period effect did not significantly impact mortality rates except for in 2004 in China, where the RR was 0.863 (95% CI = 0.764–0.974). Except for in 2009, the period effect was significant in the USA, and the RR continuously increased from 0.619 (95% CI = 0.403–0.951) in 1994 to 1.707 (95% CI = 1.113–2.618) in 2019.

When analyzing the cohort effects, we observed that earlier birth cohorts had a higher risk of PCa compared with later cohorts in both China and the USA, with the RR in China continuously decreasing from 7.722 (95% CI = 4.393–13.575) in the 1904–1908 cohort to 0.23 (95% CI = 0.005–10.315) in the 1999–2003 cohort, and the RR in the USA decreased from 6.898 (95% CI = 5.076–9.375) in the 1904–1908 cohort to 0.182 (95% CI = 0.012–2.65) in the 1999–2003 cohort. The cohort effect was significant within China for the earlier cohort of 1934–1938, and for the earlier cohorts of 1939–1943 and cohorts between 1964–1968 and 1989–1993 in the USA. In both China and the USA, earlier birth cohorts had higher mortality rates compared with later cohorts, with the RR in China continuously decreasing from 5.817 (95% CI = 1.859–18.207) in the 1904–1908 cohort to 0.129 (95% CI = 0.544–1.571) in the 1999–2003 cohort, and with the RR in the USA decreasing from 8.795 (95% CI = 3.355–23.056) in the 1904–1908 cohort to 0.12 (95% CI = 0.118–9.473) in the 1999–2003 cohort. The cohort effect was significant for the earlier cohorts of 1934–1938 and 1939–1943 in China and the USA, respectively.

## Discussion

As the most populous developing country in Asia, China has maintained an upward trend in the incidence of PCa recently, which is consistent with the results for most Chinese research cohorts (14, 15). It is therefore necessary to introduce measures to slow or reverse this trend, such as by implementing PCa screening and identifying high-risk groups. As the country with the earliest and widest implementation of PCa screening, the USA provides an effective reference when considering the impacts of PCa screening on disease burden (4). In addition, PCa occurrence and development is clearly driven by age-related characteristics and is affected by various social and environmental factors. The age-period-cohort model helps to identify high-risk ages, periods, and birth cohorts, which is greatly significant for preventing and controlling PCa. Our study indicated that people over 55 years old have a greater risk of the onset of PCa in China. This part of the population should be paid more attention for PCa prevention and control.

The incidence of PCa is significantly lower in China than in the USA and the worldwide average. The reasons relate to great differences in race-based PCa risk genes, living environments, and diets (16–20). However, with the continued increase in PCa incidence in China (e.g., the incidence in 2019 was double that in 1990), the incidence among different age groups has also increased. Meanwhile, the incidence in the USA is decreasing, particularly among those older than 70 years. These differing trends are consistent with the results obtained by Teoh et al. who studied the PCa trends in Western and Asian countries from 1988 to 2007 (21). This is partly due to the early implementation of PCa screening, which caused a decrease in PCa incidence in the USA to some extent, while gradual westernization—which causes a loss of cultural protective factors, and development of diagnostic methods (22, 23) may induce an increasing trend in China (24). Opportunistic screening has a certain impact on the incidence of PCa in China, but it should be small. According to domestic literature, <15% of male residents in large and medium-sized cities over the age of 50 have received PSA examinations, and even fewer in rural areas (25). The PCa mortality rate in the USA has also significantly decreased, which is partly related to the screening because early screening may effectively reduce PCa mortality (26). We therefore predict that implementing PCa screening in China may contribute to long-term decreases in the incidence and mortality of PCa.

The PCa incidence in China may be worse than currently reported. The literature has pointed out that in China, PCa patients younger than 56 years are often excluded from screening or misdiagnosed (27). The main reason is that no perfect screening mechanism or active monitoring mechanism is available, and doctors pay less attention to young PCa patients. Large-scale serum PCa screening in the Changchun province of China during 1998–2000 indicated that PCa prevalence in China was higher than expected. It has also been reported that screening can detect more early-stage cancers (28). On the other hand, the future situation of PCa in China is not optimistic. PCa occurs most commonly in old age, and increases 2-fold after 70 years of age in Asian countries (29). China is rapidly transforming into an aging nation. It is predicted that in 2050 there will be a large explosion in the elderly population, with up to 400 million aged above 65 years old (30). A consequence of the substantial demographic change is a surge in the prevalence and incidence of age-associated diseases encompassing PCa (31).

In addition to the increasing PCa incidence in China, its high grade at diagnosis and poor survival are also unfavorable features. The proportion of early localized PCa among newly diagnosed patients in China is only 42%, 28% have developed local progression, and 30% have distant metastases. However, in the USA, limited PCa cases accounted for 81%, lymph node metastasis cases accounted for 12%, and distant metastasis cases accounted for only 4% (32). On the other hand, studies have indicated that Asian-Pacific people are more likely to have high-grade PCa than white American, and this characteristic is not attributable to the later stage of the diagnosis (33–35). This indicates that Asian males may have biological differences that increase their susceptibility to more-serious diseases (34). Another study indicated that poorly differentiated PCa cases are more common in China than in the USA and Japan (36). The mortality-to-incidence rate ratio is also somewhat higher in China than the Asian average and much higher than the North American average (8), and the survival time of patients is short in China, with more than 27% of males dying within 5 years of a diagnosis (37). These conclusions suggest the necessity of early PCa screening in China.

In the USA, the recommendations for PSA screening have undergone many changes, resulting in unstable trends in stage-specific PCa. However, after USPSTF recommending against PSA-based screening for PCa for all men except for surveillance purposes in those with a prior PCa diagnosis in 2012, a stage “reverse migration” appeared, with a decrease in the diagnosis of localized PCa and arise in the diagnosis of locally advanced and metastatic disease reported in 2017 (38, 39). Meanwhile, the PCa mortality also increased in 2015 to 2019 as observed in this study. These adverse effects inversely confirm the significance of screening for reducing disease burden and mortality. Combined with the actual situation in China that most patients have already undergone regional or distant metastases at the time of diagnosis, only through population census can early detection of PCa and provide patients with a chance for radical treatment.

There is obvious age effect for PCa incidence and mortality as expected. However, the pattern of risk changes is different between China and the USA. In the USA, the high-risk incidence population appears in the age group over 50 years old, and the 70–74-year-old population reaches peak risk, and then decreases with the growth of the age. In China, the high-risk population appears in the age group over 55 years old, and the age group with the highest risk is 80–84 years old, delayed by 10 years compared with the USA. The age effect of mortality also shows the same pattern. This may be related to the advancement of the age of diagnosis due to the large-scale screening policy in the USA (40).

There is no significant difference in the period and cohort effects between China and the USA. The PCa incidence rates in China in 2009, 2014, and 2019 were higher than the average of the observed six periods. This may be due to the rapid economic development, social modernization, and gradual westernization of lifestyles in China, and may be closely related to improvements in diagnostic technology. This study also found that the birth cohort impacts the PCa incidence and mortality with a decreasing trend. This may be because later birth cohorts received better education than did earlier cohorts, and therefore had a stronger awareness of health and disease prevention. Due to other relevant national policies, the late birth cohort is also less exposed to PCa risk factors such as tobacco and industrial pollutants (41). Over time, more PCa risk factors have been discovered (42–44), which enhances public awareness of PCa.

Our research was subject to certain limitations. Firstly, the GBD estimates were reconstructed based on a large number of sources with different qualities, which (to some degree) may deviate from the actual data, and therefore must be validated through nationwide epidemiological surveys. Secondly, in GBD database, the cancer mortality was estimated using the cause of death ensemble model established through vital registration system data, cancer registry incidence data, and verbal autopsy data (45). The older men were more likely to suffer complicated underlying diseases, as well as limited treatments available to elderly PCa patients, resulting in that they were prone to misjudge as the PCa caused death. In other words, the mortality rate of the elderly may be overestimated. Thirdly, the parameter estimates generated from the intrinsic estimator method of age-period-cohort model are not intuitive. Meanwhile, the theoretical basis of this method is complicated, and the practical significance of parameter estimates cannot be explained. In addition, for the Joinpoint regression analysis, the existing joinpoint detection methods were based the grid search which are computationally demanding, and so, the maximum number of computable joinpoints is limited. Fourthly, information on the clinical staging of PCa was not included in the analyzed database, and so we could not determine the epidemiological trends of high-grade PCa.

## Conclusions

This study has revealed that the incidence of PCa is increasing in China while it is decreasing in the USA. Trends in disease incidence and mortality in the USA suggested screening may be beneficial to control the burden of disease in China. In addition, the age, period and cohort effects of the incidence and mortality of PCa in China may provide certain references for the formulation of screening policies.

## Data Availability Statement

The raw data of this study can be found though the Global Health Data exchange software (http://ghdx.healthdata.org/gbd-2019).

## Author Contributions

JL and HH: study design. LL and DH: data collection. HH and FX: data analyses. HH: manuscript writing. JL: manuscript proofing. All authors: results interpretations. All authors contributed to the article and approved the submitted version.

## Funding

This study was supported by the National Social Science Foundation of China (No. 16BGL183) and the Science and the Key Research and Development Program of Shaanxi Province, China (No. 2019SF-140). The funders had no role in the study design, collection, analysis, interpretation or writing of the manuscript.

## Conflict of Interest

The authors declare that the research was conducted in the absence of any commercial or financial relationships that could be construed as a potential conflict of interest.

## Publisher's Note

All claims expressed in this article are solely those of the authors and do not necessarily represent those of their affiliated organizations, or those of the publisher, the editors and the reviewers. Any product that may be evaluated in this article, or claim that may be made by its manufacturer, is not guaranteed or endorsed by the publisher.
